# Energetics and structure of grain boundary triple junctions in graphene

**DOI:** 10.1038/s41598-017-04852-w

**Published:** 2017-07-06

**Authors:** Petri Hirvonen, Zheyong Fan, Mikko M. Ervasti, Ari Harju, Ken R. Elder, Tapio Ala-Nissila

**Affiliations:** 1 COMP Centre of Excellence, Department of Applied Physics, Aalto University School of Science, P.O. Box 11000, FIN-00076 Aalto, Espoo Finland; 2 Department of Physics, Oakland University, Rochester, Michigan 48309 USA; 3 Department of Mathematical Sciences and Department of Physics, Loughborough University, Loughborough, Leicestershire LE11 3TU UK

## Abstract

Grain boundary triple junctions are a key structural element in polycrystalline materials. They are involved in the formation of microstructures and can influence the mechanical and electronic properties of materials. In this work we study the structure and energetics of triple junctions in graphene using a multiscale modelling approach based on combining the phase field crystal approach with classical molecular dynamics simulations and quantum-mechanical density functional theory calculations. We focus on the atomic structure and formation energy of the triple junctions as a function of the misorientation between the adjacent grains. We find that the triple junctions in graphene consist mostly of five-fold and seven-fold carbon rings. Most importantly, in addition to positive triple junction formation energies we also find a significant number of orientations for which the formation energy is negative.

## Introduction

In three-dimensional (3D) polycrystalline materials, a grain boundary triple junction, or a triple junction for short, is a line defect where three grains and grain boundaries meet. In 2D materials such as graphene – the 2D allotrope of carbon discovered experimentally in 2004^1^ – the triple junctions are reduced to point defects, connected by a network of 1D grain boundaries. Triple junctions are present in all polycrystalline materials and can influence their physical properties in a number of ways. First, triple junctions have a finite formation (free) energy *f*_p_ associated with them (as compared to the pristine ground state), influencing the thermodynamics of microstructure evolution. In addition, limited triple junction mobility can result in drag on the motion of the grain boundaries connected during grain growth^2–4^. Furthermore, theoretical works indicate that fractures are likely to initiate at triple junctions^5–9^ affecting the mechanical strength of polycrystalline materials. Triple junctions can also serve as channels of improved mobility for diffusion^10–12^.

There has been some controversy regarding the energetics of triple junctions. Gibbs argued that the formation energy of triple junctions between fluid phases could be negative^13^. Later, McLean claimed that triple junction formation energies should be positive in crystalline materials^14^. King viewed triple junctions as defects to grain boundaries in analogy to grain boundaries being defects in the crystalline state^15^. From this viewpoint one can treat the grain boundaries and triple junctions as separate elements with different formation energies. Using this approach, Srinivasan *et al*. actually found negative triple junction formation energies for FCC crystals by employing the simple Lennard-Jones potential^16^. In contrast, Constantini *et al*. obtained only positive formation energies for more general triple junctions in silicon^17^. Caro and Van Swygenhoven criticised the idealization of Srinivasan *et al*. and proposed an alternative description where the grain boundaries and the triple junctions comprise a “defect matter” phase of uniform formation energy density^18^. Accounted for in this way, all triple junction formation energies were found positive in nickel. Later works have reported both positive^19, 20^ and negative^21^ values for copper and iron, respectively. To our knowledge, triple junction formation energies in realistic 2D materials have not been investigated previously – neither experimentally, nor theoretically.

To fully understand the microscopic structure and energetics of triple junctions it is crucial to study the properties of triple junctions using realistic model systems, but constructing such configurations is not a trivial task even *in silico*. Typically the procedure of initialising defect structures such as grain boundaries for atomistic calculations involves multiple steps including, for example, iterative grain growth, annealing and quenching^22^, or applying coincidence site lattice theory and optimising using force-field calculations^23^. To this end, we have recently presented a new powerful multiscale modelling strategy^24^ based on using the phase field crystal (PFC) models^25–28^ to initialise and relax atomic configurations that can then be used as input to further relaxation either with classical interaction potentials or quantum-mechanical density functional theory (DFT). In particular, we carried out a comprehensive study of the structure and formation energy of grain boundaries in graphene and showed that quantitatively accurate results can be obtained with this strategy, and the standard PFC model^25^ is well-suited for creating structurally correct grain boundaries.

The PFC models have already been exploited to investigate the role of triple junctions in grain shrinking in 2D where they were found to act as sinks to reacting dislocations^29^. However, for triple junction formation energies and structures in graphene there are no detailed experimental or theoretical data available. In this work we harness our multiscale modelling strategy to investigate the structure and the formation energy of triple junctions in graphene as function of the misorientation angle between the adjacent grains. We first use the PFC models to construct relaxed atomic level configurations of triple junctions and then refine these by carrying out further analysis with Molecular Dynamics (MD) simulations and DFT. Our results confirm the somewhat controversial existence of triple junctions with negative formation energy for several misorientations in graphene.

This paper is structured as follows: In Section II, we first detail our methodology for modelling and analysing triple junctions, and finally report and discuss the related results. More specifically, formation energies of triple junctions and their structures will be treated in Sections II D and II E, respectively. Section III gives a summary and discussion. Section IV details the PFC, MD and DFT methods used in this work.

## Results

### Model systems

**Figure 1:**
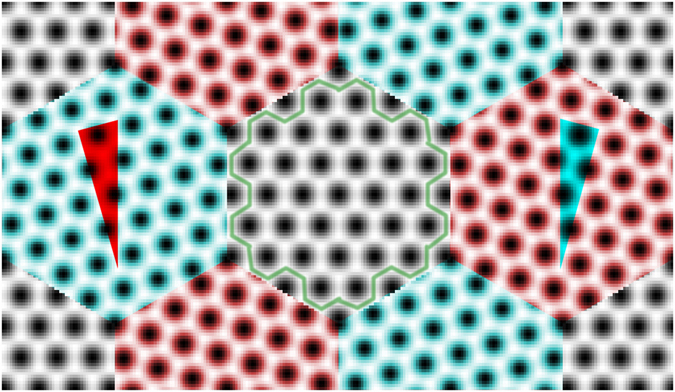
A layout of the triple junction model system. There are six grains, 12 triple junctions, six symmetric grain boundaries (between two rotated grains), and 12 asymmetric grain boundaries (between a rotated and an unrotated grain). The alternatingly rotated grains have been highlighted in red and cyan, and the rotation angles are further indicated by the red and cyan wedges. The unrotated grains at the center and at the corner have not been coloured, but the armchair (AC) edges of the former have been sketched out in green. The rotation angle here is *θ*_*AC*_ ≈ 16.1°. Note also that the periodicity of the symmetric grain boundaries between red and cyan grains exactly matches the hexagon side length. The total width of the system is about 4.6 nm.

Triple junctions come with multiple orientational and translational degrees of freedom^15^, considerably complicating their analysis. To simplify the picture we focused on a highly symmetric family of triple junctions. First, the grain boundaries that meet at the junction have 120° angles between them and, second, two of the surrounding grains are rotated symmetrically in the opposite directions from a reference orientation retained by the third grain. Figure 1 demonstrates our model system layout. The periodic 2D computational unit cell is divided into six regular hexagon-shaped and equal-sized grains leaving 12 triple junctions between them. Each model system also contains six symmetric and 12 asymmetric grain boundaries. The symmetric boundaries are located between two rotated grains (red-cyan boundaries in Fig. 1), whereas the asymmetric boundaries lie between a rotated grain and an unrotated one (red-gray and cyan-gray). While this layout exhibits two different kinds of grain boundaries, all the triple junctions are identical. We investigate the formation energies of the asymmetric grain boundaries in Supplementary Section S1 to explicitly demonstrate that PFC describes them properly, too (our previous work considered only the symmetric ones^24^). We note that a similar layout was exploited in ref. 7 where the fracture behaviour of graphene was considered.

The hexagonal grains are initialised with a honeycomb lattice using the one-mode approximation^30^

1 $$\psi (x,y)=\,\cos \,(qx)\,\cos \,(qy/\sqrt{3})-\cos \,(2qy/\sqrt{3})/2,$$

where the wave number *q* controls the length scale. Around each triple junction two grains are rotated by angles *θ* and −*θ*, where 0° < *θ* < 30° and the limits give armchair and zigzag edges, respectively. The third grain is kept fixed in a reference orientation with either armchair or zigzag edges. We use the shorthands *θ*_AC_ and *θ*_ZZ_ to indicate the reference orientation alongside the rotation angle. As a further remark, a six-fold symmetric rotation centre of the lattice is aligned to coincide with the centre point of each grain, and for the rotated grains the lattice is rotated about the grain centre.

We required that the periodicity of the symmetric grain boundaries matches the side length of the grains. We also required that the side length for the unrotated grains should be between (*n* + 0.05) a*, where *n* is an arbitrary positive integer and a* is the lattice constant for both the zigzag (a* = *a*) and armchair $({a}^{\ast }=a\sqrt{3})$ reference orientations. The systems in the present calculations have a total width $W\gtrsim 14\,{\rm{nm}}$ to avoid finite size effects that were analysed in Appendix C in ref. 24. However, we allowed for two exceptions that have *W* ≈ 9.2 nm (≈1800 carbon atoms) but are feasible to be studied using DFT. Both systems have *θ* ≈ 16.1° (corresponding to the special tilt angle *θ*_II_ in ref. 24) but different reference orientations.

Due to the highly symmetric initial state and the fact that the PFC models exhibit Peierls barriers for dislocation motion but lack thermal fluctuations, motion of the grain boundaries and triple junctions is limited within a few atomic spacings in most cases. We carefully inspected the relaxed systems and rejected all cases where the grain boundaries had become significantly curved or the triple junctions had migrated from their initial positions.

### Decomposition of formation energy

For any finite system in 2D, the total free energy *F* can be written as ref. 31

2 $$F={f}_{{\rm{s}}}A+{f}_{{\rm{L}}}L+{f}_{{\rm{p}}}N,$$

where the first term is the contribution of the pristine single crystal state, given by its surface free energy density *f*_s_ times the total area *A*. The second term is the contribution of the 1D line defects, given by the mean formation energy of grain boundaries per unit length, or the grain boundary energy *f*_L_ times the total length of the boundaries *L*. The third term is the contribution of 0D triple junctions, given by their mean formation energy *f*_p_ times their number *N*. Note that while the triple junctions considered here are macroscopically identical, they are likely to display small differences in structure and energy due to the inevitable breaking of symmetry during numerical relaxation. The same applies for the two types of grain boundaries as well^24^.

The formation energy with respect to the single-crystalline ground state Δ*F* can be obtained by calculating the energy of the ground state independently and by subtracting it from Eq. (2) which for the PFC models reads

3 $${\rm{\Delta }}F=F-{f}_{{\rm{s}}}A={f}_{{\rm{L}}}L+{f}_{{\rm{p}}}N.$$

For the atomistic MD and DFT calculations the corresponding expression is given by

4 $${\rm{\Delta }}F=(\epsilon -{\epsilon }_{{\rm{eq}}})\,{N}_{{\rm{C}}}={f}_{{\rm{L}}}L+{f}_{{\rm{p}}}N,$$

where $\epsilon$ and ${\epsilon }_{{\rm{eq}}}$ denote the energy per carbon atom in a defected system and in the pristine state, respectively, and *N*_C_ is the number of carbon atoms.

The model systems have *N* = 12 and $L=2\sqrt{3}W=6H$, where *W* and *H* are the width and height of a system. By scaling the system dimensions, *f*_L_ and *f*_p_ can be solved as the slope *d*(Δ*F*)/*dL* and as one-twelfth the intercept Δ*F*(*L* = 0), respectively. The width of the systems is given by

5 $$W=m{W}_{0}\,(\theta ),$$

where *m* = 1, 2, 3, 4, 5 and *W*_0_ gives, for a particular *θ*, the smallest width for which the constraints detailed above in Section II A hold. Note that *W*_0_ is different between the two reference orientations. For all cases of *θ* considered in this study, we tabulate the corresponding values of *W*_0_ in Supplementary Table S2. Due to using a system size optimization algorithm (see Sec. IV A), the final *W* and *H* varied a little from their initial values, but only negligibly even for small *m*. For the MD and DFT systems, we estimated *L* using the dimensions of the original planar PFC1 configurations and took into account the small differences in the equilibrium lattice constants by rescaling *L* → (*a*_i_/*a*_PFC1_) *L*, where *a*_i_ (i = MD, DFT) and *a*_PFC1_ are the equilibrium lattice constants given by either MD or DFT, and PFC1. Using this approach, one can estimate *L* consistently without needing to worry about the out-of-plane buckling of the monolayer.

As pointed out by an anonymous referee, an alternative to the scaling analysis described above is to vary *N* while keeping *L* fixed. We compared the two approaches and found consistent results. The alternative scaling analysis is presented in Supplementary Section S3. We thank the anonymous referee for the helpful comment.

### Scaling analysis

**Figure 2:**
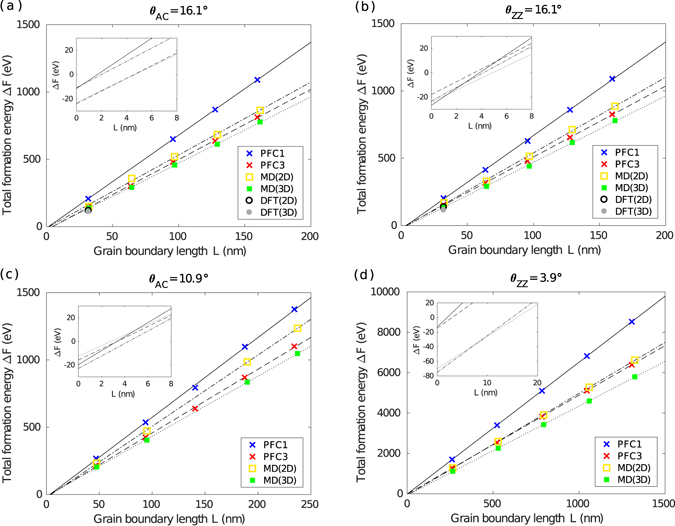
Scaling of the total formation energy for triple junction model systems. Data are given by PFC1, PFC3, MD and DFT as a function of the total grain boundary length *L*. The atomistic configurations were either constrained to a plane (2D), or allowed to relax freely in three dimensions (3D). The straight lines are linear fits to the data. The insets magnify the limit *L* → 0 to which the data have been extrapolated. The systems corresponding to (**a**–**d**) have *θ*_AC_ ≈ 16.1°, *θ*_ZZ_ ≈ 16.1°, *θ*_AC_ ≈ 10.9°, *θ*_ZZ_ ≈ 3.9° and *W* ≈ 9.2 *m*, 9.2 *m*, 14 *m*, 75 *m* nm, respectively. Note that the two cases with the smallest *W*_0_ depicted in (**a**,**b**) have the same *θ* but different reference orientations.

Figure 2 demonstrates the scaling of the total formation energy Δ*F* as a function of the total grain boundary length *L* for some rotation angles and reference orientations. Results are given of PFC1, PFC3, MD and DFT calculations. Linear fits (least squares) to these data are also given whose slope and intercept indicate the mean grain boundary energy and the total triple junction formation energy, respectively. One-sigma confidence intervals for the estimated slope and intercept are used to obtain all error estimates in the subsequent figures. Of the cases presented, (a) and (b) correspond to the two cases with the smallest *W*_0_ whose analysis is feasible using DFT for *m* = 1. Panel (c) corresponds to the case with the third smallest *W*_0_, and (d) to one of larger system sizes.

As anticipated based on Eqs (3) and (4), all plots in Fig. 2 show excellent linear scaling. In the whole of the PFC1, PFC3, MD(2D) and MD(3D) data, *R* ^2^ > 0.999 for all the cases except for a few PFC and MD cases where *R* ^2^ > 0.996. The total formation energy increases with length, approximately 5 eV per nanometre of grain boundary, which is consistent with our previous results for the grain boundary energy of symmetric boundaries (<8 eV/nm)^24^ and with those of earlier works referenced therein. In the insets to the panels we show the extrapolated limit of *L* → 0 magnified. The intercepts are in all cases very close to zero when compared to the absolute energy scale, but it can already be seen here that in some cases the intercepts are negative. For such cases, the crossover from negative to positive total formation energy typically occurs where *L* is only a few nanometres, corresponding to nanometre-wide systems that cannot be realised with the present layout. Even in the most extreme cases, the width required is much smaller than *W*_0_.

In these plots and in our data in general, PFC3 agrees quite well with both MD(2D) and MD(3D). MD(3D) gives values lower than MD(2D), because the corresponding systems are allowed to relax further by buckling out of plane^32^. We carried out DFT calculations to validate our PFC and MD results. The energies given by DFT(2D) and DFT(3D) are expectedly low – the two DFT data points correspond to systems where the symmetric grain boundaries have a particularly low energy; see the kink at 2*θ* ≈ 32.2° in Fig. 4 of ref. 24. Nevertheless, DFT, MD and PFC3 are all in good agreement. This was to be expected based on our previous work^24^. On the other hand, the total formation energy given by PFC1 rises noticeably steeper. This is not surprising because PFC1 has been shown to overestimate grain boundary energies^24^.

**Figure 3:**
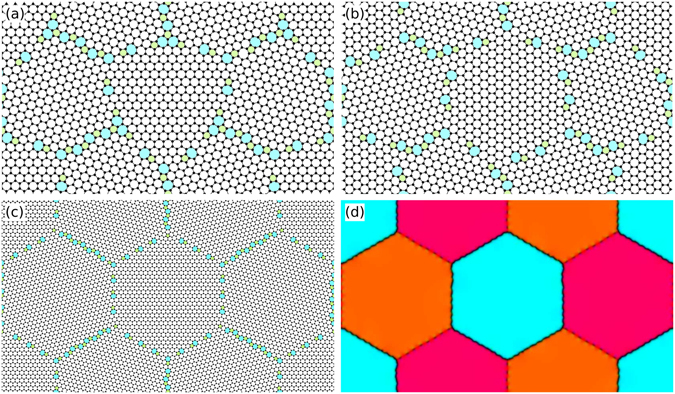
Some typical relaxed triple junction configurations. Panels (a) and (b) show the two smallest DFT(2D) systems corresponding to Fig. 2(a,b), respectively, with *m* = 1. The pentagons and heptagons are highlighted in green and in cyan, respectively. (**c**) A larger version of (**a**) with *m* = 2 and relaxed using PFC1. (**d**) A coarse-grained representation of the PFC1 system corresponding to Fig. 2(d) with *m* = 1. Coarse graining has been done by filtering out the atomic level structure and by mapping the local crystallographic orientation to different hues; see comment [60] in ref. 24 for details. The chains of dark dots are individual dislocations along small-angle armchair grain boundaries, and the slightly undulating solid black lines are large-angle armchair-zigzag boundaries.

Figure 3 demonstrates some of the configurations corresponding to the cases shown in Fig. 2. The systems in panels (a) and (b) correspond to the two smallest systems with *m* = 1. These systems have been relaxed using DFT(2D) and have retained the topology of the initial PFC1 configuration. In both cases, the hexagonal grain structure is identifiable but far from ideal. Despite the distortion of the hexagonal structure, the formation energies of these two systems line up nicely with those of their larger *m* counterparts, as can be seen in Fig. 2(a,b). Panel (c) showcases a larger version of the system portrayed in (a) with *m* = 2 and relaxed using PFC1. While the linear system size is only doubled, the grain boundaries are highly ordered and the hexagonal layout is almost ideal. The same applies to the larger PFC1 system in panel (d) as well that corresponds to Fig. 2(d) with *m* = 1. Note that the asymmetric grain boundaries in (d) demonstrate some intrinsic undulation, but, in the macroscopic limit, they can be viewed as straight, mathematically thin lines.

### Formation energy of triple junctions

#### Overview

**Figure 4:**
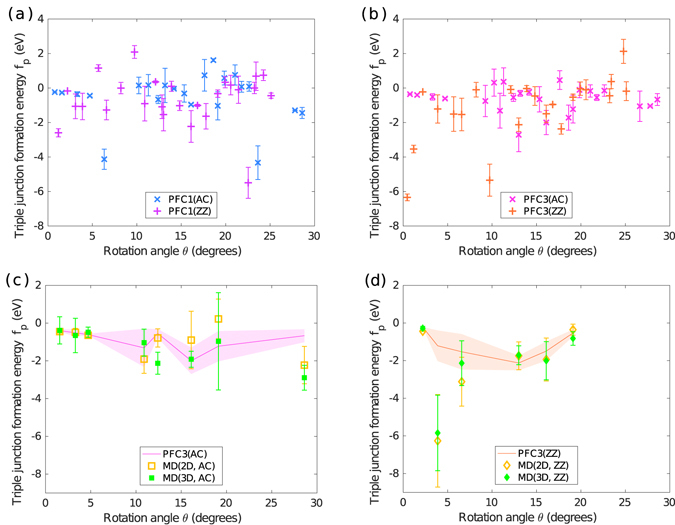
Triple junction mean formation energy *f*_p_. Data are given by (**a**) PFC1, (**b**) PFC3 and (**c**,**d**) PFC, MD(2D) and MD(3D) as a function of the rotation angle *θ*. Subfigures (**a**,**b**) offer data of both armchair (AC) and zigzag (ZZ) reference orientations, whereas (**c**,**d**) treat these limits separately. In (**c**,**d**), PFC3 results of cases not studied using MD have been omitted. Furthermore, in (**c**,**d**), the PFC3 data points included and their error estimates have been plotted with solid lines and lighter-coloured bands, respectively, to alleviate the overlap of data markers and error bars. Note that the lines and the bands are not indicative of the triple junction energy between the data points.

Figure 4 gives the mean formation energy per triple junction calculated using PFC1, PFC3 and MD as a function of *θ*. Panels (a) and (b) focus on PFC1 and PFC3, respectively. Panels (c) and (d) compare PFC3 to MD for both reference orientations separately. We chose to use PFC3 over PFC1 in this comparison because it has been found to describe the formation energy of grain boundaries more accurately^24^. PFC data points with error estimates $\gtrsim$1 eV per triple junction have been omitted as unreliable. Furthermore, data are lacking for *θ*_ZZ_ ≥ 25° due to imperfect relaxation of the systems. This is elaborated further in Supplementary Section S4.

The PFC1 and PFC3 data shown in Fig. 4(a,b), respectively, display both positive and negative formation energies with their average slightly on the negative side – roughly −0.5 eV for both models. Most of the data show no obvious trends, but for *θ*_AC_ < 5°, both models give a consistent set of four data points with small error margins and slightly negative formation energies decreasing with increasing *θ*. In this limit, both the symmetric and asymmetric grain boundaries are small-angle boundaries sparse with dislocations. At the triple junctions, therefore, only the elastic fields of the surrounding dislocations come together. With increasing *θ*, complexity grows as dislocations meet at the triple junctions and this trend vanishes. In general, the microscopic arrangements of dislocations in the model systems are so complex that a systematic mapping between the structures and the features in the formation energy falls beyond the scope of this work. Nevertheless, we investigated fitting sums of sinusoidal wave modes to the triple junction energy to reveal possible periodic trends in the data, but did not find clear evidence of periodicity.

The data in Fig. 4(a,b) are not clearly divided between the armchair and zigzag reference orientations; rather, the two datasets appear to follow each other to some extent. While the correlation is not perfect and the data hold significant uncertainty, this modest agreement suggests that the formation energy is dominated by misorientation rather than by the microscopic differences between triple junctions of the two reference orientations.

The absolute scale of the formation energies is very low, of the order of a few electron volts for both models. For the most part, this is lower than has been reported for isolated defects in graphene: 5 eV^33^ and 7.5 eV^32^ for a 5|7 dislocation, and 7.6 and 4.8 eV for a vacancy and a Stone-Wales defect, respectively^34^. Previous experimental and theoretical studies have also reported low triple junction line energies in 3D materials. Fortier first measured a relatively high value of 3 keV/nm^35^, but more recent works^17–21^ have reported significantly lower estimates of the order of $\lesssim$40 eV/nm. If a triple junction in graphene is viewed as a line defect of length 0.35 nm, the results of these more recent works correspond to absolute values of $\lesssim$14 eV per triple junction.

#### Details of the energetics

The striking fact that can be seen in our results is that there are indeed many misorientation angles where the triple junction formations energy is negative. To check that this indeed is the case, we used MD simulations to verify the low formation energies obtained. Unfortunately, in cases where the PFC models give the most negative energies, the corresponding system sizes are too large to obtain converged MD results in a reasonable time. Nevertheless, as shown in Fig. 4(c,d), our MD results corresponding to moderately negative PFC data points support negative formation energies. For both the armchair and zigzag reference orientations the agreement between PFC3, MD(2D) and MD(3D) is fairly good with significant overlap and just a few outliers. The MD(2D) and MD(3D) results show minor differences between them, and in most cases the MD(3D) values are lower as expected. In cases where the MD(2D) values are lower, the corresponding results fit within each other’s error margins. At *θ*_ZZ_ ≈ 3.9° both MD(2D) and MD(3D) give particularly low formation energies around −6 eV, but with large error margins.

If one compares the data given by the different methods, one finds a few interesting data points either in a particularly good agreement or in a strong disagreement. Two examples of agreement are found at *θ*_AC_ ≈ 4.7° and *θ*_ZZ_ ≈ 2.2°. For these two cases, all methods predict slightly negative triple junction formation energies with small error margins. In the former case, all grain boundaries have small misorientations and are, therefore, sparsely populated with dislocations. There are no defects at the triple junction core – only overlapping elastic fields of the surrounding dislocations. The latter case displays symmetric boundaries of small misorientation and asymmetric ones with a large misorientation. As a consequence, there are two grain boundaries packed tightly with dislocations and a sparse one that all coincide at the triple junction. The small error bars are indicative of triple junction and grain boundary structures that are highly similar for all *m*.

At *θ*_AC_ ≈ 17.6°, both PFC models agree on a data point whose energy exceeds that of the neighbouring data points. A closer inspection of the triple junction structures revealed defect topologies that appear similar to those corresponding to the neighbouring data points. As we will discuss in Section II E, there are microscopic differences between triple junctions both within a single model system and in systems of different *m*. In this case, for PFC1 model systems with *m* > 3, we observe isolated heptagons (see Section II E) at some triple junctions, whereas for *m* < 4 we do not. It is possible that for this particular *θ* isolated heptagons are energetically preferred and bias the fitted scaling line to yield a higher formation energy for the triple junctions.

On the other hand at *θ*_ZZ_ ≈ 9.7°, PFC1 and PFC3 disagree strongly with the former showing a positive formation energy and the latter giving a clearly negative one. Both methods have preserved the initial hexagonal layout perfectly during relaxation. The grain boundaries in the systems are straight and appear identical at mesoscopic scales. At the atomic level, however, PFC3 systems display tetragons^24^ near some of the triple junctions. It is likely that the rise in formation energy is due to the presence of such metastable tetragons. The case at *θ*_ZZ_ ≈ 24.8° also shows a relatively high formation energy according to PFC3, but PFC1 predicts a slightly negative value. At a larger scale, the hexagonal layout and the grain boundaries in the corresponding PFC systems appear to have relaxed without issues. However, an atomic level inspection of the PFC3 systems again reveals various unexpected structures near the triple junctions, such as carbons with four bonds, dimers^26^ and many others. Also in this case metastable structures are the probable culprit for the higher value of energy.

#### Origin of the negative formation energies

Based on our analysis we can conclude that negative formation energies are abundant in graphene. Because the triple junctions here are likely to display small microscopic differences to one another, the values reported here must be viewed as average formation energies per triple junction. Because of the high-symmetry initial state, many of the triple junctions have very similar atomic-level structures and formation energies.

The possibility of negative formation energies has been criticised because they could endlessly multiply destabilising the material. King^15^, however, gives arguments against such a scenario. First of all, triple junctions can exist only in conjunction with grain boundaries whose formation energy is always positive (by definition). Based on the low absolute values determined for triple junction energies in this work, and on the relatively large grain boundary energies obtained in our previous work^24^, the grain boundaries dominate the total formation energy. Second, if a triple junction were to multiply, its orientational variables would not, in general, be identical and the anisotropy is likely to result in an increase in the total formation energy. Furthermore, a triple junction does not necessarily have defects in its core – especially if it links three small-angle grain boundaries together – in which case it is merely a region where the elastic fields of the grain boundary segments composed of sparse chains of dislocations can either add up or cancel, resulting in an energy contribution that is either positive or negative, respectively.

Lastly, we would like to note, that treating triple junctions as zero-dimensional elements, with the constant part of the total energy as their formation energy, is the only mathematically rigorous definition, because it makes no assumptions of the spatial distribution of energy. While triple junctions could, in principle, be treated as finite-size elements, with always positive formation energies^18^, such approaches must assume unrealistic energy distributions with step-like or other arbitrary profiles.

### Structure of the triple junctions

#### PFC configurations

Despite the fact that the PFC3 calculations were initialised with relaxed PFC1 configurations, they resulted in varying topologies for the grain boundaries as already discussed in our previous work^24^. While many of the PFC3 triple junctions contain only pentagons and heptagons, alternative structures such as tetragons and octagons, as well as structures too ambiguous for topological reconstruction, are also observed. Therefore, we decided to limit the topological analysis to the more realistic PFC1 structures with pentagons and heptagons only.

**Figure 5:**
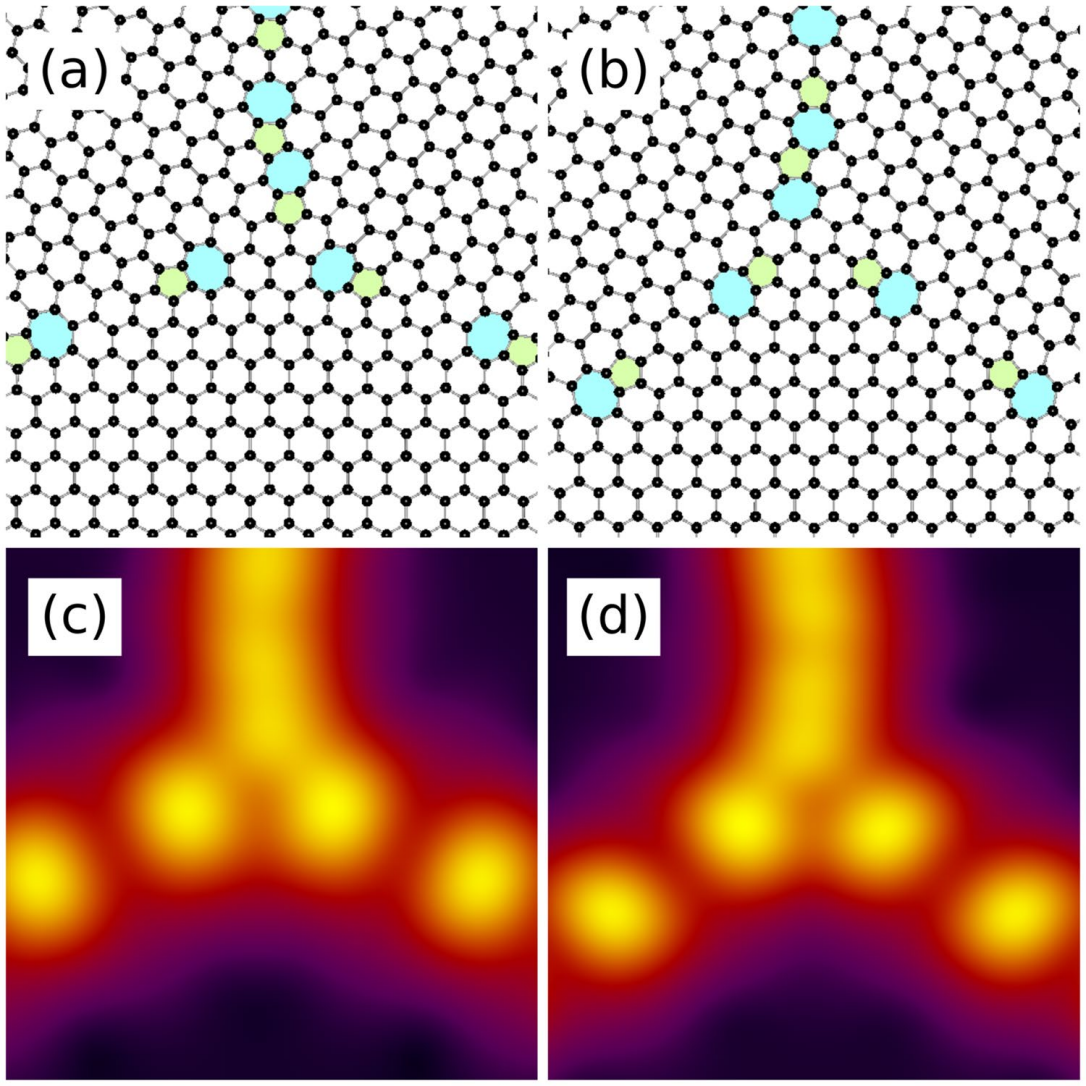
Two seemingly similar triple junctions in a single system. In panel (a) there is a pentagon and two heptagons facing the triple junction, and *vice versa* in (**b**). Panels (c) and (d) give the smoothed free energy density (see Supplementary Note S5) in (**a**,**b**), respectively. Here *θ*_AC_ ≈ 13.2°.

As mentioned, most of the model systems retained the high degree of symmetry of the initial state during relaxation, and display highly periodic arrangements of dislocations along their grain boundaries. Nevertheless, a closer inspection reveals some differences in the atomic-level structure between different triple junctions within the same model systems. Figure 5 juxtaposes two triple junctions found within the same model system that appear similar. Note however, that the dislocations present in (a) are facing the opposite direction with respect to the ones in (b). As a result, (a) has one pentagon and two heptagons facing the triple junction, whereas in (b) the orientations are reversed. Geometry necessitates that such asymmetry is always present in our model systems. The corresponding smoothed free energy density plots (see Supplementary Note S5) shown in Fig. 5(c,d) appear identical, suggesting very similar formation energies for the two alternate structures.

**Figure 6:**
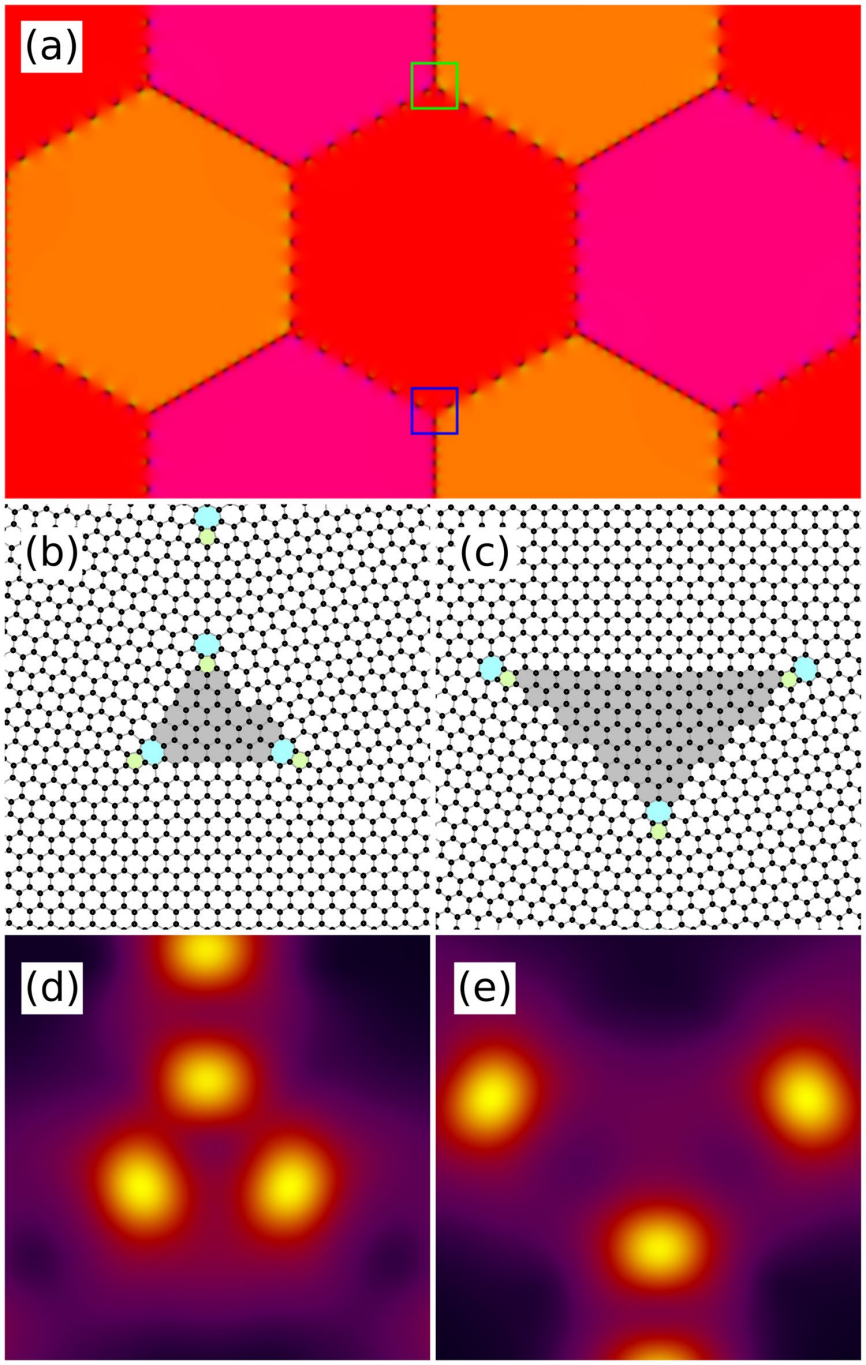
A model system with more and less crowded triple junctions. The system (**a**) has two types of triple junctions where the dislocations are either closer together (**b**) or farther apart (**c**). The corresponding smoothed free energy densities are shown in (**d**,**e**), respectively. The green and blue boxes in (**a**) indicate where the junctions shown in (**b**,**c**), respectively, are located. In (**b**,**c**) the triangles spanned by the dislocations are shaded gray to better illustrate the degree of separation. Here, *θ*_AC_ ≈ 4.7°.

Figure 6 demonstrates another case of topologically different triple junctions. Here, the system shown in panel (a) the dislocations come close together around every second triple junction; compare with (b) and (c). In graphene, the strain energy fields of dislocations decay ∝1/*r* ^4^ ^33^ which can result in a significant difference in formation energy between the two junctions. Panels (d) and (e) visualise the smoothed free energy density and further demonstrate the difference in overlap between the elastic fields.

**Figure 7:**
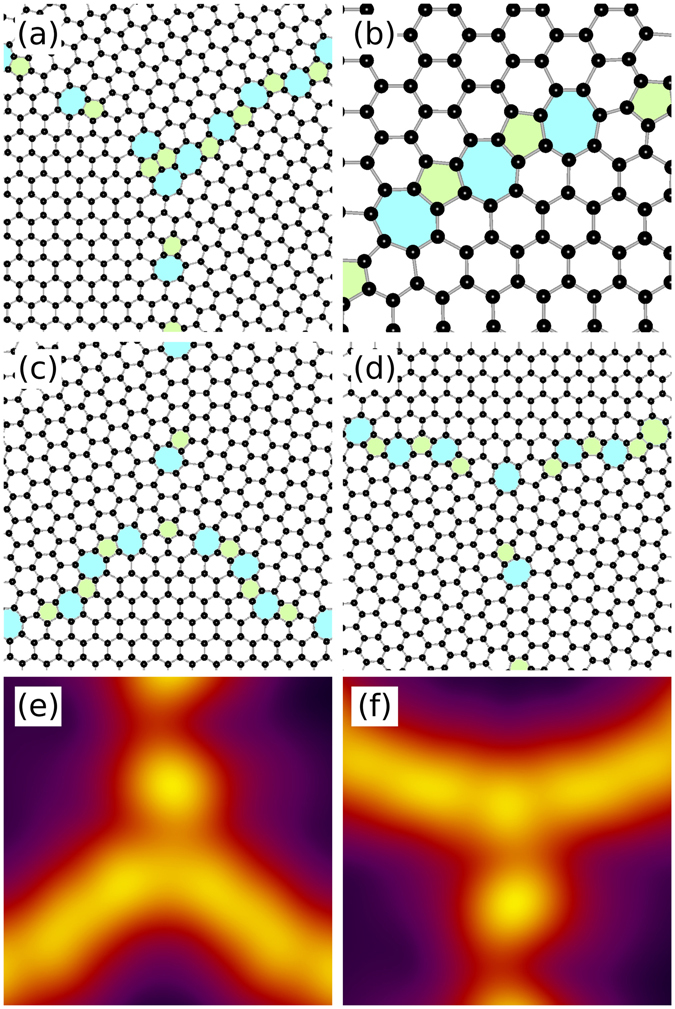
More complicated defect structures at and near the triple junctions. (**a**) A cluster of 5|7 dislocations at a triple junction (*θ*_AC_ ≈ 16.1°). (**b**) A chain of coalesced 5|7 dislocations terminating with heptagons at both ends (*θ*_ZZ_ ≈ 0.8°). (**c**) An isolated pentagon and (**d**) an isolated heptagon at triple junctions within the same model system (*θ*_AC_ ≈ 25.5°). Panels (e) and (f) give the smoothed free energy density of the triple junctions shown in (**c**,**d**).

In the absence of large angle grain boundaries, the only defects observed at the triple junctions are 5|7 dislocations. However, when large angle boundaries are involved, more complex arrangements of pentagons and heptagons are also observed that were not encountered in isolated grain boundaries^24^. Examples of such structures are given in Fig. 7.

In triple junctions, the dislocations can form clusters where two or more like polygons are connected, whereas in isolated low-energy PFC1 grain boundaries pentagons are not connected to other pentagons and similarly heptagons not to other heptagons^24^. Figure 7(a) demonstrates two pairs of connected pentagons and heptagons. In large-angle PFC1 grain boundaries the 5|7 dislocations coalesce to form continuous chains of alternating pentagons and heptagons, terminating with a pentagon at one end and with a heptagon at the other^24^. At triple junctions, as well as along grain boundaries in their vicinity, we observe very short chains with same kind of polygons at both ends; see Fig. 7(b). In addition, isolated pentagons and heptagons surrounded solely by hexagons are relatively common as well; see Fig. 7(c,d), respectively. The distribution of free energy density shown in (e) and (f) is similar in the vicinity of both isolated non-hexagons, but the heptagon results in a peak distinguishable in the smoothed free energy density. In most of the close-ups presented in Fig. 7, the angles between the grain boundaries appear to deviate somewhat from 120°, but this is explained by intrinsic undulation of the asymmetric boundaries; recall the undulation of the asymmetric boundaries in Fig. 3(d).

The results presented in this section highlight the fact that not all of the triple junctions within a model system are identical. The microscopic structural variations inevitably reduce the analysis to that of average triple junction formation energies with finite statistical error.

#### DFT calculations

We used DFT(3D) calculations to verify the stability of the different types of PFC1 triple junction topologies as discussed earlier in this section. To facilitate this task, we cut out from the model systems small, roughly circular flakes centred around the triple junctions. We prepared these flakes with about 300–400 atoms to ensure quick numerical convergence. The edges of the flakes were passivated with hydrogen atoms and no boundary constraints were imposed.

**Figure 8:**
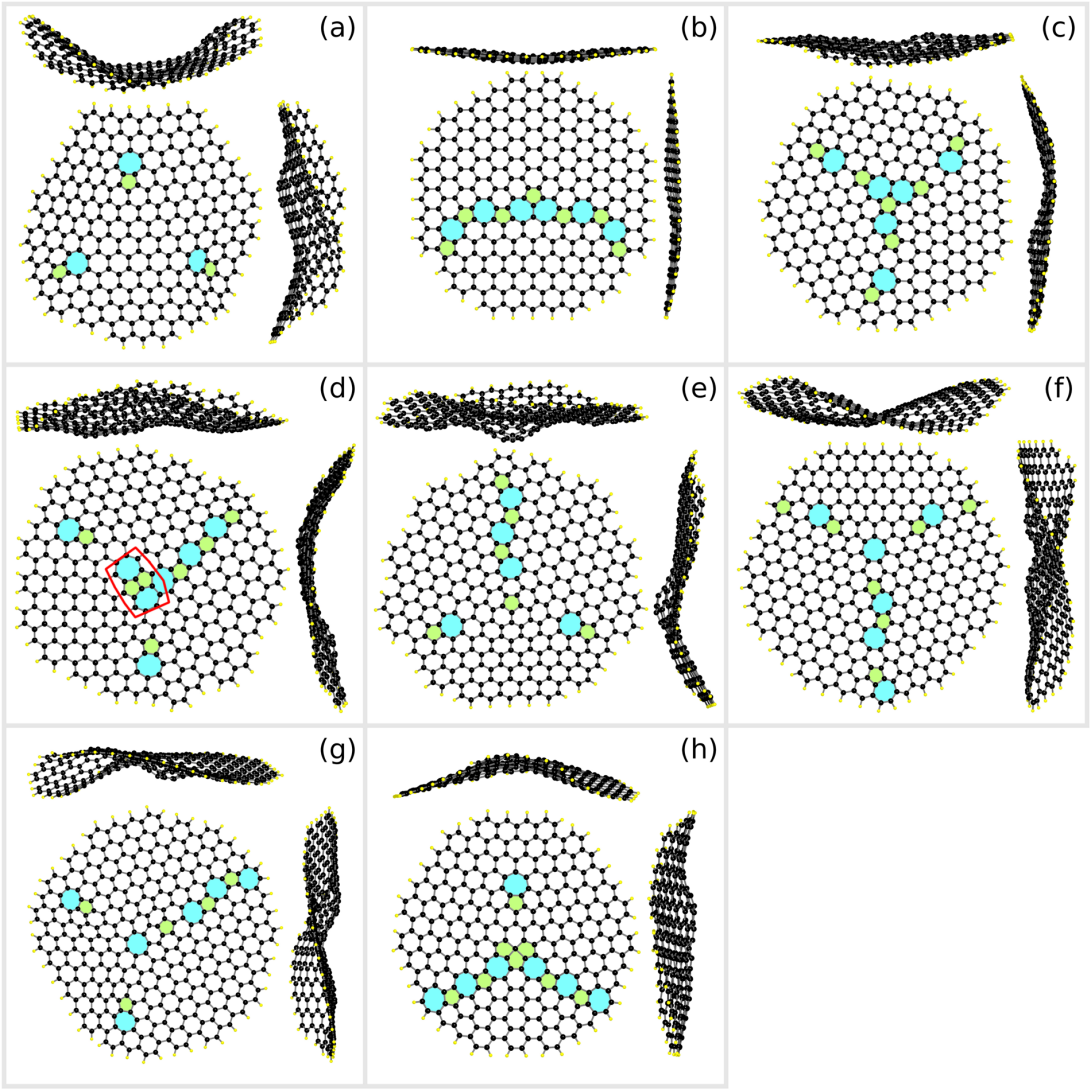
Small flakes with various triple junction structures. The flakes have been relaxed using DFT(3D) with hydrogen-passivated edges (yellow atoms) and free boundary conditions. On top and adjacent to each flake are side views as seen from the corresponding sides. The flakes in (**a**–**g**) have been extracted from a PFC1 system, whereas the one in (**h**) is from a PFC3 system. (**a**) A triple junction with three low-angle boundaries and no core defect (*θ*_AC_ ≈ 4.7°). (**b**) A pair of heptagons connected to each other (*θ*_AC_ ≈ 27.8°). The third, small misorientation grain boundary had a dislocation spacing greater than the flake radius. (**c**) A pair of heptagons connected to each other (*θ*_ZZ_ ≈ 10.9°) with a third grain boundary of a larger misorientation. (**d**) Two opposite 5|7 dislocations (*θ*_AC_ ≈ 16.1°). A closed Burgers loop is sketched in red. (**e**) An isolated pentagon (*θ*_AC_ ≈ 16.1°). (**f**) An isolated heptagon (*θ*_AC_ ≈ 16.1°). (**g**) An isolated pentagon and an isolated heptagon (*θ*_AC_ ≈ 16.1°). (**h**) Three connected pentagons (*θ*_ZZ_ ≈ 5.7°).

Figure 8 displays a collage of triple junction flakes relaxed using DFT(3D). All of the flakes investigated retained their initial topology predicted by PFC during relaxation. The PFC1 flakes depicted in (a)–(d) demonstrate increasing levels of clustering of pentagons and heptagons in the triple junction core. The three isolated 5|7 dislocations in (a) remained stable during relaxation and kept from slipping and annihilating each other. Stability of the fairly simple triple junction structures in (b) and (c) may not come as a surprise, but in (d) there are two opposite 5|7 dislocations with a net Burgers vector of zero that one might expect to annihilate each other. However, the two pentagons in the middle cannot fuse into a hexagon due to the two excess carbons between them that would have to be transported elsewhere. No other reactions take place, either. The PFC1 flakes in (e)–(g) display isolated pentagons and heptagons. In the vicinity of isolated nonhexagons the surrounding honeycomb lattice must deviate from its six-fold rotational symmetry which necessitates dislocations around this region and considerable elastic energy^33^ – a recipe for structural transformations. However, all of these triple junction structures were found to be stable. The flake (h) with three connected pentagons was extracted from a PFC3 model system. With the introduction of an extra carbon, the outer two of the three connected pentagons could transform into hexagons, leaving a chain of pentagons and heptagons. However, with the number of carbons fixed, this structure proved stable.

The side views in Fig. 8 reveal that while the topologies extracted from planar PFC systems are stable, the triple junction structures tend to buckle out of plane. We must point out, however, that only qualitative conclusions can be drawn from the buckling of such small flakes. Nevertheless, it is well known that grain boundaries themselves can give rise to significant curvature and folding of the monolayer^23, 33, 36–41^, and are likely to give the major contribution to the buckling observed here as well. However, the isolated pentagon in Fig. 8(e) results in a distinct conical protrusion in the buckled sheet. Previously^33^, a conical out-of-plane profile has been verified for flakes occupied by a solitary pentagon. Similar buckling is visible also in (g) due to the isolated pentagon. The isolated heptagons in (f) and (g) also result in a significant local curvature. Surprisingly, the cluster of three pentagons in panel (h) gives rise to relatively small local curvature in the sheet; rather, the right-hand side view reveals that greatest curvature is located around the isolated 5|7 dislocation. Buckling at triple junctions will be investigated in more detail in a future work.

## Summary and Discussion

In this work we have employed an efficient multiscale protocol^24^ to model the energetics and atomic structures of triple junctions in graphene. We first use phase field crystal (PFC) models to initialise relaxed atomic configurations in 2D, which are then mapped into atomic coordinates for further relaxation with classical MD and quantum-mechanical DFT calculations both in 2D and 3D. We exploit a highly symmetric layout for our model systems to ensure consistent results. We concentrate here on the formation energy of triple junctions as a function of the misorientation between the adjacent grains. Our calculations show that there indeed exist both positive and negative formation energies on the order of a few electronvolts. This energy scale is low compared to the formation energy of the grain boundaries, which dominates the total energy of the systems. We consistently find slightly negative formation energies for triple junctions with small-angle armchair grain boundaries, but no obvious trends beyond this.

In addition, we have studied the atomic-level structure of the triple junctions. We investigate the subtle microscopic differences between different triple junctions and, moreover, explored the different types of defect topologies possible at a triple junction core. In addition to 5|7 dislocations clustered to varying degrees and in different arrangements, isolated pentagons and heptagons were discovered as relatively common structures. Using DFT(3D) calculations, we have verified the stability of the different types of topologies predicted by PFC, and also showed that the out-of-plane curvature of the monolayer varies significantly between different triple junction structures.

## Methods

### Phase field crystal models

PFC is a relatively new approach for modelling microelasticity in crystalline materials^25^. PFC models describe the structure of matter at the atomic scale via a smooth classical density field $\psi ({\boldsymbol{r}})$. The systems modelled using PFC are governed by a free energy functional $F[\psi ({\boldsymbol{r}})]$ that is minimised when $\psi$ is constant or periodic. While the former case can be viewed as corresponding to a disordered (liquid) phase, the latter corresponds to crystalline states whose symmetries can be matched with a crystal structure of interest via the choice of *F*. In addition to equilibrium crystal structures, defect structures with arbitrary crystal shapes, positions and orientations are possible, and elastoplastic processes ranging from dislocation slip to evolution of large-scale microstructures can be captured. The numerically convenient nature of PFC models and their standard relaxational dynamics give access to largely uncharted multiscale modelling regimes that combine large length and time scales with atomic-level spatial features. As a consequence, PFC is particularly well-suited for modelling the structural and mechanical properties of microstructures^24^.

In our previous work^24^ we compared four different PFC models in terms of the topology and the formation energy of symmetrically tilted grain boundaries to find the best model for graphene. We found that the simplest of the models – the standard one-mode PFC model (PFC1) – well captures the topology of graphene grain boundaries with realistic chains of 5|7 dislocations^42^, whereas the three-mode model (PFC3) is in good quantitative agreement with the atomistic techniques DFT and MD in terms of the formation energy of the grain boundaries. We will use both of these models here. The free energy of the PFC1 model is given by

6 $${F}_{1}={c}_{1}\int d{\boldsymbol{r}}(\frac{\psi { {\mathcal L} }_{1}\psi }{2}+\frac{\tau {\psi }^{3}}{3}+\frac{{\psi }^{4}}{4}),$$

where

7 $${ {\mathcal L} }_{1}=\epsilon +{({q}_{0}^{2}+{\nabla }^{2})}^{2},$$

and that of the PFC3 model

8 $${F}_{3}={c}_{3}\int d{\boldsymbol{r}}(\frac{\psi { {\mathcal L} }_{3}\psi }{2}+\frac{{\psi }^{4}}{4}+\mu \psi ),$$

where

9 $$\begin{array}{rcl}{ {\mathcal L} }_{3} & = & \epsilon +\lambda ({b}_{0}+{({q}_{0}^{2}+{\nabla }^{2})}^{2})\,({b}_{1}+{({q}_{1}^{2}+{\nabla }^{2})}^{2})\\  &  & \times ({b}_{2}+{({q}_{2}^{2}+{\nabla }^{2})}^{2}).\end{array}$$

The free energies above combine a double-well potential $\epsilon {\psi }^{2}/2+{\psi }^{4}/4$ with gradient terms that favour a spatially oscillating $\psi$ with certain length scales. The odd-powered terms break the symmetry between the two double-well minima and thereby control the average density, which in part controls the stability of the 2D honeycomb phase. Of the parameters, *c*_1_ and *c*_3_ are coefficients for selecting the energy scale of the models, $\epsilon$ is related to temperature, *λ* and *b*_*j*=0,1,2_ weight the contributions of the modes given by *q*_*j*=0,1,2_, and *τ* and *μ* control the asymmetry of the double-well potential.

We use the standard nonconserved dynamics for efficient equilibration of the model systems. Further details about the dynamics, the numerical method used and the model parameter values are given in ref. 24. The system size optimization algorithm described in Appendix B in ref. 24 was used in all PFC calculations to eliminate global strain effects.

### Molecular dynamics

The triple junctions and the accompanying grain boundaries were investigated using a in-house GPUMD code implemented fully on graphics processing units (GPUs)^43–45^. We used the Tersoff potential^46^, but with optimised parameters provided by Lindsay and Broido^47^ that are well suited for modelling graphene. We performed MD simulations at a low temperature of 1 K in order to suppress the entropic contribution to the formation energy. Furthermore, the in-plane stress was set to zero in a barostat^48^. We adopted the velocity-Verlet integration method^49^ with a time step of 1 fs. For each system, a simulation time of 2 ns is used to ensure full convergence of the data.

### Density functional theory

Since DFT involves a much larger numerical effort than either PFC or MD, we studied the formation energies and structures of only the very smallest triple junction model systems. We used the all-electron FHI-aims package^50^ with numerical atom-centred basis functions to ensure that the grain boundary regions are treated accurately. The default *light* basis sets were employed together with the GGA-PBE functional^51^. Since the periodic computational cells were large (roughly 1800 carbon atoms), only the ${\rm{\Gamma }}$ point was included in the *k* grid. During the course of the calculations, the self-consistent cycle was considered converged if, among other things, the total energy had converged down to 10^−6^ eV between consecutive iterations. The computational cells and the atomic geometries were relaxed in each case until the forces acting on the atoms were smaller than 10^−2^ eV/Å.

### Multiscale approach

We investigated the formation energy of grain boundary triple junctions by varying the misorientation between the grains. In our previous work^24^, the PFC1 model was found efficient and robust for constructing realistic atomic configurations. Therefore, here we also used PFC1 to equilibrate our model systems and to estimate the formation energy of the triple junctions. The relaxed PFC1 configurations were used as the starting point for comparative PFC3 and DFT calculations, and MD simulations. The procedure of converting a continuous PFC density field into atomistic coordinates for the calculations is described in ref. 24. For the initialization of PFC3 calculations, the equilibrated PFC1 density fields were renormalised to match the average density, amplitude and lattice constant of the PFC3 ground state.

### Data availability

The PFC systems generated and analysed during the current study are not publicly available due to their large size, but other numerical data and visualization are available from the corresponding author on reasonable request.

## Electronic supplementary material

Supplementary information
