# Supplementary material for: Energetics and structure of grain boundary triple junctions in graphene
Source: Sci Rep. 2017 Jul 6;7:4754. doi: 10.1038/s41598-017-04852-w (PMC5500591; doi:10.1038/s41598-017-04852-w)
Supplement: Supplementary file 1 — Supplementary information [file 41598_2017_4852_MOESM1_ESM.pdf]

Energetics and structure of grain boundary triple junctions in graphene

Petri Hirvonen,[1,*] Zheyong Fan,[1] Mikko M. Ervasti,[1]
Ari Harju,[1] Ken R. Elder,[2] and Tapio Ala-Nissila[1,3]

[1]*COMP Centre of Excellence, Department of Applied Physics,*
Aalto University School of Science,
P.O. Box 11000, FIN-00076 Aalto, Espoo, Finland

[2]*Department of Physics, Oakland University, Rochester, Michigan 48309, USA*

[3]*Department of Mathematical Sciences and Department of Physics,*
Loughborough University, Loughborough, Leicestershire LE11 3TU, UK

[*] email: petri.hirvonen@aalto.fi

S1. FORMATION ENERGY OF ASYMMETRIC GRAIN BOUNDARIES

We have previously studied the formation energy of symmetric grain boundaries [1], but because they are a special case of all the possible grain boundaries linked by triple junctions, we demonstrate here that PFC models can be used to describe also the asymmetric boundaries present in our systems. The system layout contains six symmetric and 12 asymmetric grain boundaries whose contributions can be decomposed into two components as

$$f_{\mathrm{L}} L = \gamma_{\mathrm{s}} \frac{L}{3} + \gamma_{\mathrm{as}} \frac{2L}{3}, \tag{S1}$$

where $\gamma_{\mathrm{(a)s}}$ is the mean grain boundary formation energy of (a)symmetric boundaries. In our previous work [1] we presented a detailed analysis of grain boundary energies calculated for symmetric boundaries using PFC, DFT and MD. Because we know γ_{s}, we can write

$$\gamma_{\mathrm{as}} = \frac{3 f_{\mathrm{L}}}{2} - \frac{\gamma_{\mathrm{s}}}{2}. \tag{S2}$$

Note that we did not limit ourselves to the same misorientation angles as in our previous work [1], whereby the values for γ_{s} were estimated by linear interpolation from the corresponding sets of lowest energy grain boundaries reported therein. Linear interpolation is justified, as these data are smooth and are accurate. We verified the correctness of our results for γ_{as} by carrying out a small set of independent bicrystal calculations using both PFC1 and PFC3, where we considered $\theta_{\mathrm{AC,ZZ}} \approx 10^\circ, 20^\circ$ and 30°. We found consistent results with the relative error in $\gamma_{\mathrm{as}} \lesssim 2\%$ between triple junction and bicrystal calculations.

Figure S1 shows the mean grain boundary energy of asymmetric boundaries γ_{as} as given by PFC1, PFC3 and MD(3D) as a function of the misorientation angle between the rotated and unrotated grains. Data from previous DFT [2] and MD [3] studies are also presented for comparison. Results are given for both armchair and zigzag reference orientations, and are plotted in panels (a) and (b), respectively. The rotation angle θ is also indicated in both panels by a secondary horizontal axis. In Section II A in the main article, we chose $\theta = 0^\circ$ for armchair and $\theta = 30^\circ$ for zigzag edges in the rotated grains. For the zigzag reference orientation this means that the misorientation angle is given by $30^\circ - \theta$.

In Fig. S1 (b) data are lacking for small misorientation boundaries due to imperfect relaxation of the systems. This is elaborated further in Supplementary S4. Furthermore, MD(2D) results have been omitted, because in our previous work [1] where symmetric boundaries

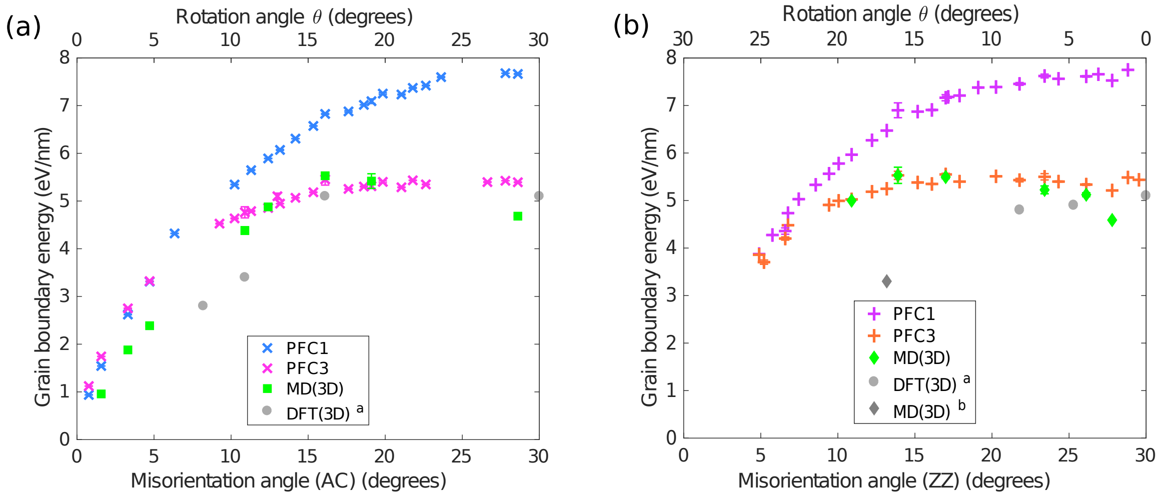

FIG. S1. Grain boundary energy of asymmetric grain boundaries. Data are given by PFC1, PFC3 and MD(3D) as a function of the misorientation angle between the rotated and unrotated grains. Data are also given from Refs. [2][a] and [3][b]. (a) Results for the armchair (AC) and (b) for the zigzag (ZZ) reference orientation. The secondary horizontal axis indicates the rotation angle θ.

were studied, we used a different potential for calculating the corresponding γ_s, a prerequisite for γ_{as} in Eq. (S2).

In Fig. S1, the grain boundary energy saturates towards the maximum misorientation of $\theta = 30°$, as expected. The results are very similar between both reference orientations which indicates that misorientation clearly dominates the formation energy over the microscopic details of the boundaries. Furthermore, no dips indicating highly ordered low-energy boundaries are visible in the data. This is in contrast to our previous work on symmetric grain boundaries [1] where two dips corresponding to well-known low energy boundaries were clearly visible. The error bars for the data points are mostly very small.

PFC1 traces a roughly parabolic grain boundary energy trend peaking close to 8 eV/nm. The curve from PFC3 data is flatter for larger misorientations and saturates above 5 eV/nm. Our MD(3D) values come somewhat lower for small misorientations, as well as near the maximum misorientation of 30°, but nevertheless agree well with PFC3. In general, the present grain boundary energies are very similar to those found previously for symmetric boundaries of same misorientation [1] – a further proof that the energy is controlled mainly by misorientation. The data from previous works [2, 3] is consistent with our results for misorientations exceeding 15°, but below this there is significant discrepancy – these results

suggest roughly $(1-2)$ eV/nm lower energies around the approximate misorientation of 10°. These data are from 3D DFT [2] and MD [3] studies. Based on our previous work [1], we would have expected lower DFT energies at large misorientations. A possible explanation is that the corresponding grain boundary defect configurations are not in the ground state.

S2. TABLE OF RESULTS

TABLE S1: The rotation angles θ and system widths W_0 considered, and the triple junction mean formation energies obtained using PFC1 (f_p^{PFC1}), PFC3 (f_p^{PFC3}), MD(2D) ($f_p^{\mathrm{MD(2D)}}$) and MD(3D) ($f_p^{\mathrm{MD(3D)}}$). The first 26 lines of data are for the armchair reference orientation and the rest for zigzag. The values for W_0 assume a lattice constant of 0.246 nm and are reported for PFC1. The corresponding values for PFC3 can be obtained by scaling with the relative difference of the length scales 7.33125/7.26218. The error estimates for the triple junction mean formation energies are obtained from their one-sigma confidence intervals. The missing PFC1 and PFC3 data are due to rejected data points. Cases, where data from both models are rejected, are not shown.

**Rotation θ (°)**	**Width W_0 (nm)**	$f_p^{\mathbf{PFC1}}$ **(eV)**	$f_p^{\mathbf{PFC3}}$ **(eV)**	$f_p^{\mathbf{MD(2D)}}$ **(eV)**	$f_p^{\mathbf{MD(3D)}}$ **(eV)**
0.769	190.421	-0.234 ± 0.002	-0.355 ± 0.004		
1.575	93.023	-0.265 ± 0.043	-0.402 ± 0.062	-0.448 ± 0.083	-0.385 ± 0.719
3.304	44.354	-0.354 ± 0.136	-0.513 ± 0.186	-0.493 ± 0.214	-0.657 ± 0.911
4.715	31.101	-0.444 ± 0.055	-0.609 ± 0.048	-0.645 ± 0.048	-0.486 ± 0.272
6.336	347.506	-4.126 ± 0.586			
9.249	174.967		-0.751 ± 0.908		
10.227	143.984	0.152 ± 0.480	0.313 ± 0.788		
10.893	13.528		-1.312 ± 1.010	-1.906 ± 0.753	-1.037 ± 0.714
11.302	117.405	0.164 ± 0.619	0.363 ± 0.805		
12.404	95.211	-0.666 ± 0.235	-0.544 ± 0.228	-0.788 ± 0.482	-2.127 ± 0.581
13.004	22.723		-2.709 ± 0.978		
13.174	168.264	0.154 ± 0.994	-0.304 ± 0.172		
14.176	73.073	-0.038 ± 0.027	-0.233 ± 0.181		
15.336	183.660	-0.315 ± 0.503	-0.653 ± 0.730		
16.102	9.218	-0.959 ± 0.053	-1.996 ± 0.702	-0.899 ± 1.525	-1.914 ± 0.425

TABLE S1 – continued from previous page

**Rotation** θ **(°)**	**Width** W_0 **(nm)**	$f_p^{\mathbf{PFC1}}$ **(eV)**	$f_p^{\mathbf{PFC3}}$ **(eV)**	$f_p^{\mathbf{MD(2D)}}$ **(eV)**	$f_p^{\mathbf{MD(3D)}}$ **(eV)**
17.617	92.918	0.740 ± 0.916	0.463 ± 0.541		
18.613	168.206	1.616 ± 0.082	-1.718 ± 0.730		
19.107	23.431	-1.031 ± 0.814	-1.225 ± 0.800	0.221 ± 1.048	-0.964 ± 2.579
19.842	37.660	0.579 ± 0.402	-0.106 ± 0.456		
21.052	99.638	0.760 ± 0.584	-0.170 ± 0.354		
21.787	61.992	0.075 ± 0.315	-0.549 ± 0.173		
22.635	86.355	0.089 ± 0.272	-0.147 ± 0.332		
23.624	159.490	-4.325 ± 0.974			
26.628	188.229		-1.055 ± 0.882		
27.796	115.128	-1.293 ± 0.017	-1.041 ± 0.040		
28.603	90.783	-1.443 ± 0.320	-0.668 ± 0.341	-2.226 ± 0.989	-2.894 ± 0.659
0.466	314.398		-6.337 ± 0.195		
1.181	124.010	-2.593 ± 0.239	-3.535 ± 0.221		
2.204	66.469	-0.167 ± 0.006	-0.220 ± 0.006	-0.439 ± 0.006	-0.276 ± 0.101
3.098	141.903	-1.059 ± 0.718			
3.886	75.449	-1.065 ± 0.767	-1.212 ± 0.817	-6.262 ± 2.459	-5.843 ± 2.007
5.685	129.047	1.157 ± 0.197	-1.509 ± 1.001		
6.587	22.287	-1.272 ± 0.571	-1.531 ± 0.942	-3.120 ± 1.297	-2.135 ± 1.191
8.213	17.896	-0.002 ± 0.328	-0.098 ± 0.423		
9.740	166.232	2.090 ± 0.372	-5.341 ± 0.928		
10.893	162.333	-0.904 ± 1.004			
12.103	158.503	0.360 ± 0.059	-0.081 ± 0.218		
12.839	172.578	-1.085 ± 0.683			
13.004	22.723	-1.540 ± 0.939	-2.125 ± 0.389	-1.745 ± 0.739	-1.714 ± 0.503
13.898	31.931	0.100 ± 0.295	-0.029 ± 0.174		
14.822	109.930	-1.025 ± 0.280	-0.462 ± 0.544		
16.102	9.218	-2.225 ± 0.920	-1.493 ± 0.507	-1.940 ± 1.142	-2.005 ± 1.018
16.826	97.147	-1.006 ± 0.065	-0.955 ± 0.030		

TABLE S1 – continued from previous page

**Rotation** θ **(°)**	**Width** W_0 **(nm)**	$f_p^{\mathbf{PFC1}}$ **(eV)**	$f_p^{\mathbf{PFC3}}$ **(eV)**	$f_p^{\mathbf{MD(2D)}}$ **(eV)**	$f_p^{\mathbf{MD(3D)}}$ **(eV)**
17.784	167.407	-1.636 ± 0.737	-2.369 ± 0.301		
19.107	70.292	-0.322 ± 0.214	-0.532 ± 0.182	-0.356 ± 0.294	-0.823 ± 0.362
19.946	164.870	0.333 ± 0.318	-0.037 ± 0.389		
20.570	94.591	0.172 ± 0.567	-0.101 ± 0.576		
21.435	118.926	-0.098 ± 0.787			
22.537	153.412	-5.492 ± 0.898			
23.234	187.934	0.005 ± 0.330	-0.451 ± 0.400		
23.413	19.301	0.686 ± 0.823	0.377 ± 0.412		
24.244	242.814	0.744 ± 0.307			
24.791	24.388		2.129 ± 0.696		
25.107	337.411	-0.441 ± 0.151	-0.183 ± 0.547		

S3. ALTERNATIVE SCALING ANALYSIS

The formation energy of triple junctions can also be extracted by varying the number of triple junctions N while keeping the total grain boundary length L fixed. As suggested by an anonymous referee, we determined the triple junction formation energy for 7 different rotation angles in this way. Because our PFC1, PFC3 and MD results are in good mutual agreement, we tried this alternative approach only with PFC1. Figure S2 illustrates the model system layouts considered. The base case shown in (a) is composed of 8 periodic copies, whereas in (b)-(d) the number of copies is always halved and their size doubled with respect to the previous case. In each system, the total grain boundary length is $8L$ while the total number of triple junctions decreases as $8N, 4N, 2N, N$, respectively.

Compared to the original approach where L is scaled, this approach has a weakness – it gives only 4 data points to fit a scaling curve to, but the computational effort is already multiplied. Relative model system areas for scaling L and N are 1, 4, 9, 16 and 25, and 8, 16, 32 and 64, respectively. Using the spectral method described in our previous work [1], a single relaxation step scales as $\mathcal{O}\left[n \log\left(n\right)\right]$ where n is the number of grid points ($\propto$ area),

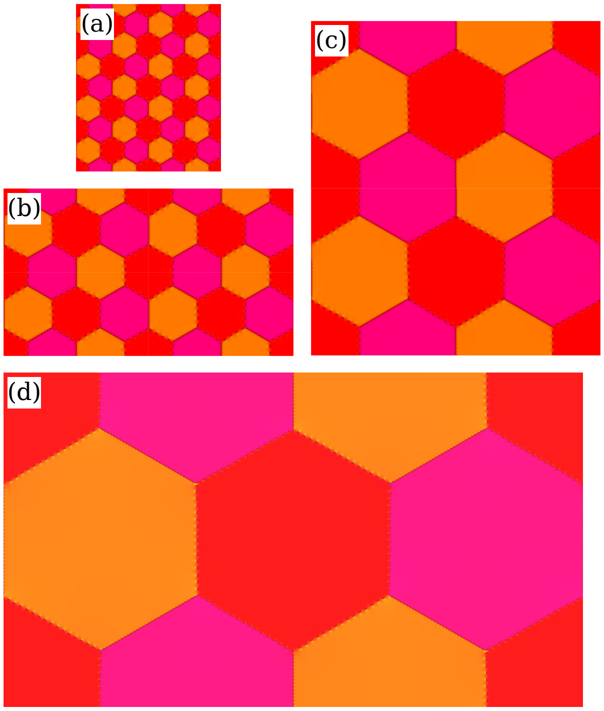

FIG. S2. Model systems for scaling the number of triple junctions N. (a)-(d) The model systems for the case $\theta_{AC} \approx 4.7°$. The crystallographic orientations of the rotated grains have been mapped to different hues. The relative sizes of the systems are to scale.

and in general, larger systems require more relaxation steps for equilibration. In view of this, we chose to study cases where the model system sizes are moderate.

Figure S3 compares the results obtained using these two approaches. Our previous data ($PFC1_L$ and $PFC3_L$ – the subscript indicates the variable varied) are contrasted with the new PFC1 values ($PFC1_N$) as a function of the rotation angle. For the most part, the formation energies are very consistent and the PFC1 results fit within each other's error bars. At $\theta_{AC} \approx 16.1°$, however, $PFC1_N$ gives a significantly higher energy. This is due to different triple junction topologies: for the largest model systems, $PFC1_N$ has produced junctions with simple chains of $5|7$ dislocations only, whereas $PFC1_L$ gave also isolated nonhexagons and more clustered $5|7$ configurations such as illustrated in section II.E.1 in the main article. Nevertheless, the overall consistent results support the results obtained by varying L, and further validate the approach originally used.

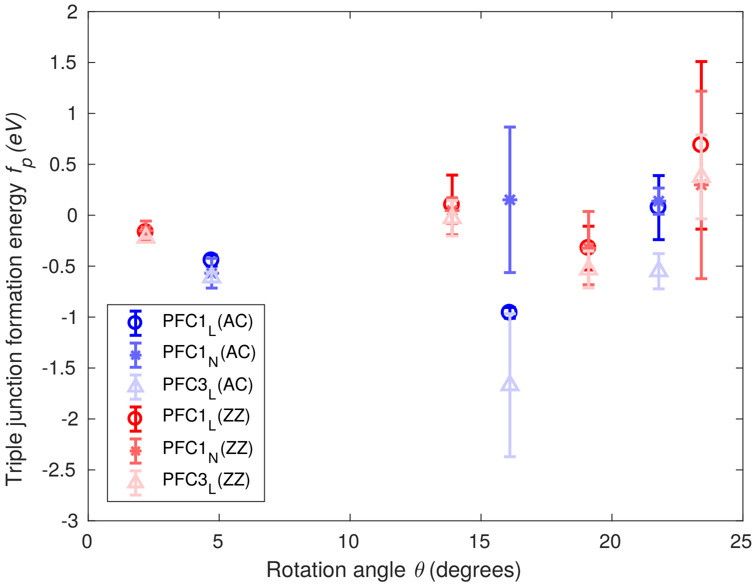

FIG. S3. Triple junction mean formation energy as a function of the rotation angle. Results are given of scaling L (PFC1$_L$ and PFC3$_L$) and of scaling N (PFC1$_N$) for both reference orientations (AC) and (ZZ).

S4. DISLOCATION TRIPLETS

In Fig. 4 (a), (b) and (d) in the main article, as well as in Fig. S1 (b), results are lacking for $\theta_{\mathrm{ZZ}} \geq 25°$ and for the corresponding small misorientation angles of the zigzag reference orientation, respectively. Inspection of the systems corresponding to this limit revealed that during the relaxation seemingly random numbers of metastable dislocation triplets have formed along the asymmetric grain boundaries instead of pairs of dislocations. It is unlikely that an existing pair of 5|7 dislocations dissociates into a triplet during relaxation; rather, the sharp interfaces preceding the asymmetric boundaries in the initial state most likely serve as perturbations that give rise to the triplets directly. While the triplets have a higher formation energy, their net Burgers vector equals that of the pairs; see Fig. S4 for further details. In principle, a dislocation pair can dissociate further into more than just three dislocations and still retain the net Burgers vector, but the high concentration of excess energy makes this unlikely and we observed at most triplets. Because the number of dislocation triplets varies for different m, the scaling of the total formation energy is too noisy for a reliable linear fit in this limit.

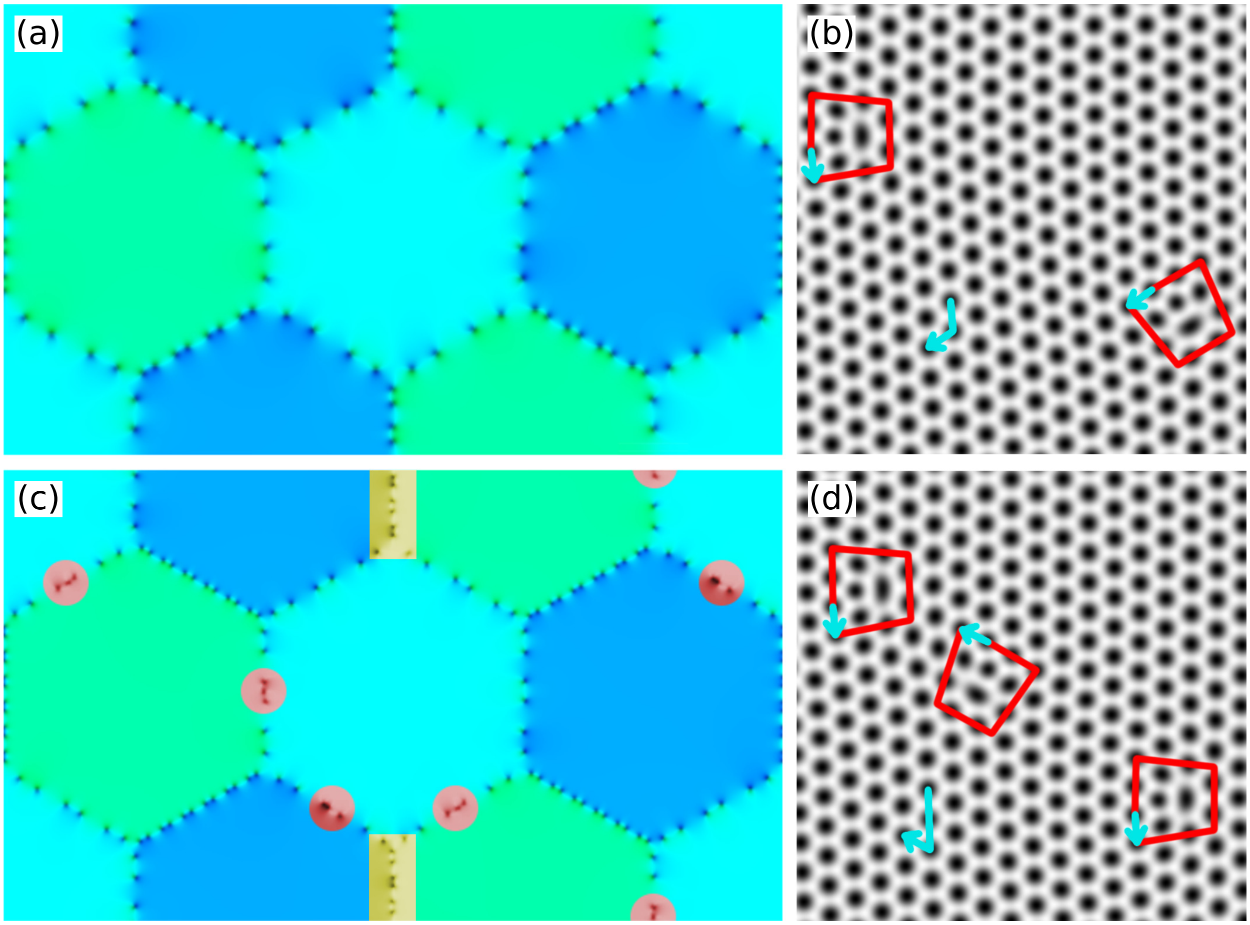

FIG. S4. Dislocation pairs and triplets along asymmetric small misorientation grain boundaries where $\theta_{ZZ} \approx 26.8°$. In the smaller system with $m = 2$ (a) no triplets are observed after relaxation, but the larger system with $m = 3$ (b) has six triplets present (highlighted in red). Note also that along the symmetric grain boundary highlighted in yellow, the distribution of dislocations is not as ideal as for the other symmetric boundaries. Panels (c) and (d) offer blow-ups of the dislocation pairs and triplets in the respective systems. Burgers loops have been sketched in red around the dislocations and Burgers vectors are indicated by cyan arrows. The net Burgers vectors are also demonstrated disconnected from the Burgers loops.

S5. VISUALIZATION OF SMOOTHED FREE ENERGY DENSITY

The periodic free energy density has been smoothed by convolving with a Gaussian kernel to highlight the excess energy of the defects. The smoothed free energy density has been normalized and then transformed by taking its fourth root to better visualize its decay $\propto 1/r^4$ around dislocations [3]. The transformed data have been mapped linearly to both

the brightness and the hue (from blue via magenta and red to yellow in the HSV color model [4]).

[1] Hirvonen, P. *et al.* Multiscale modeling of polycrystalline graphene: A comparison of structure and defect energies of realistic samples from phase field crystal models. *Phys. Rev. B* **94**, 035414 (2016).

[2] Zhang, J., Zhao, J. & Lu, J. Intrinsic Strength and Failure Behaviors of Graphene Grain Boundaries. *ACS Nano* **6**, 2704–2711 (2012).

[3] Liu, Y. & Yakobson, B. I. Cones, Pringles, and Grain Boundary Landscapes in Graphene Topology. *Nano Lett.* **10**, 2178–2183 (2010).

[4] Joblove, G. H. & Greenberg, D. Color Spaces for Computer Graphics. *Comp. Graph.* **12**, 20–25 (1978).
